# A reporter gene system for screening inhibitors of Wnt signaling pathway

**DOI:** 10.1007/s13659-012-0094-0

**Published:** 2013-01-09

**Authors:** Xing-Yao Li, Yuan-Yuan Wang, Chun-Mao Yuan, Xiao-Jiang Hao, Yan Li

**Affiliations:** 194 State Key Laboratory of Phytochemistry and Plant Resources in West China, Kunming Institute of Botany, Chinese Academy of Sciences, Kunming, 650201 Yunnan, China; 294 University of Chinese Academy of Sciences, Beijing, 100049 China; 394 School of Marine Science and Technology, Harbin Institute of Technology at Weihai, Shandong, 264209 China

**Keywords:** Wnt signaling, natural compounds, dual-luciferase reporter, tumor

## Abstract

Abnormal activation of canonical Wnt signaling has been associated with various types of cancer. Inhibitory reagents targeting the Wnt signaling have great potential to inhibit the growth of relevant tumors. Here we generated a cell-based screening strategy for identification of antagonists of the Wnt/β-catenin signaling pathway. Stable expression *wnt*3a was generated in HEK293 cell line, which harbors dual-luciferase reporters. The Wnt signaling in the stably transfected cell line was proved to be very sensitive to (−)-epigallocatechin-3-gallate (EGCG) and lithium chloride (LiCl) treatment, respectively. Natural compounds were screened and a couple of novel inhibitory modulators of the Wnt signaling pathway were obtained.

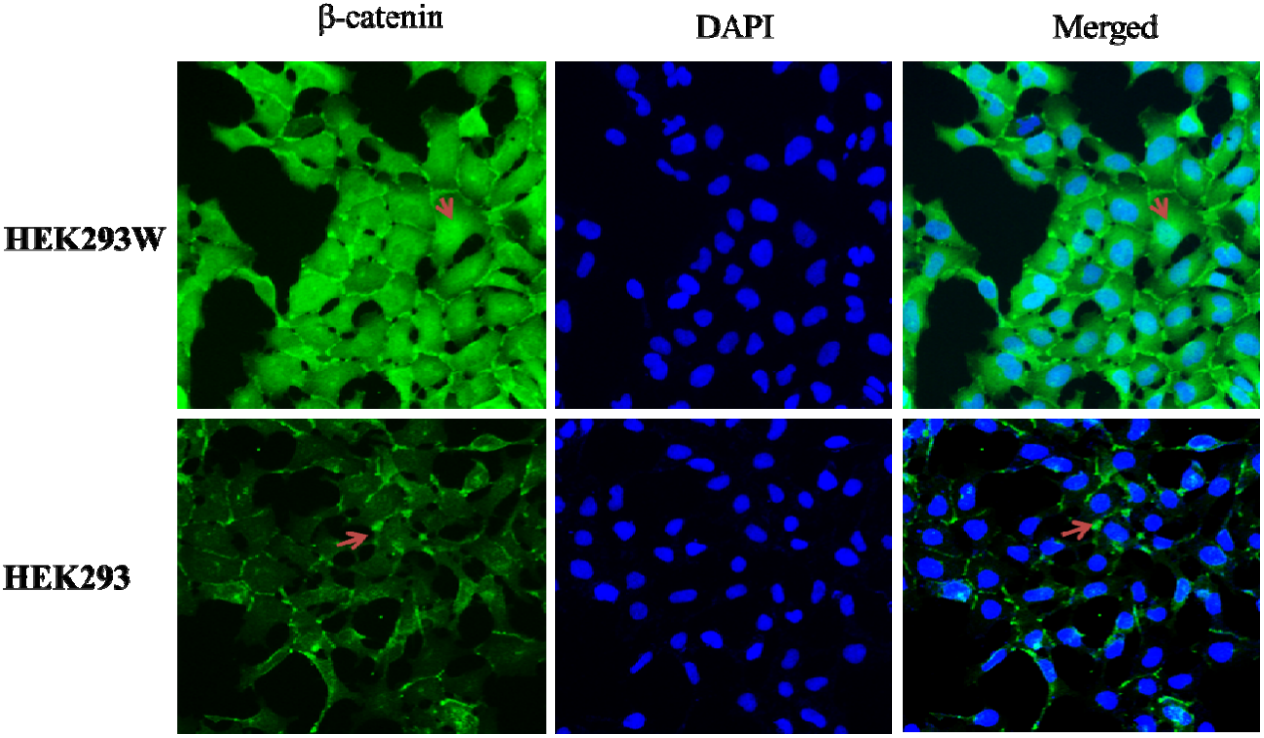

## Introduction

In the past several decades, the obvious importance of the cell signaling pathways as potential cancer therapeutic targets has stimulated researchers and pharmaceutical companies to develop numerous chemical inhibitors of these pathways like the Hedgehog and Notch signaling pathways[1]. Canonical Wnt/β-catenin pathway is essential for normal development and the maintenance of tissue stem cells[2,3]. This pathway can be activated by the binding of the Wnt ligands to its membrane receptor complex, resulting in the inhibition of the β-catenin destruction complex and subsequent trans-activation of β-catenin target genes promoting proliferation[2]. Wnt/β-catenin pathway is frequently abnormal in many types of malignancies such as colon cancer[4–6], liver cancer[7], leukemia[8,9], melanoma[10], pancreatic cancer[11], and breast cancer[12]. The activation state is due to mutations, such as oncogene β-catenin or the tumor suppressor gene APC and Axin2 in colon cancers, resulting in abrogated destruction complexes, consequently constitutive active and stabilized β-catenin proteins[13].

To explore the potential anti-tumor drug leads targeting the Wnt signaling pathway from the natural compounds, we generated a dual-luciferase reporter assay and utilized for screening the inhibitors of Wnt signaling pathway. Several inhibitory compounds were discovered and the structure-activity relationship was analyzed.

*To whom correspondence should be addressed. E-mail: liyanb@mail.kib.ac.cn (Y. Li); haoxj@mail.kib.ac.cn (X.J. Hao)

## Results and Discussion

**Generation of *wnt*3a Stably Expressed Cell Line HEK293W.** HEK293 cells were co-transfected with TopFlash, renilla reporters and *wnt*3a. For obtaining stable transfectants, several G418 and hygromycin B resistant clones were isolated. These G418 and hygromycin B resistant clones were verified by luciferase reporter assay. Figure 1 showed that 6 clones expressesed remarked TopFlash luciferase activity and no signal was detected in control HEK293 cells. Clone 6 showed

**Figure 1.** HEK293 cells were co-transfected with TopFlash, *wnt*3a and renilla plasmids. 6 clones were identified (G418 and hygromycin B resistant) by reporter assay analysis. Figure 1 showed that 6 clones expressesed luciferase activity and no signal was detected in control HEK293 cells. Clone 6 shown significantly high luciferase activity compared to other clones.

significantly high luciferase activity compared with others clones, and was named HEK293 Wnt3a-expressed (HEK293W).

**Wnt Signaling in HEK293W Cell Line was Sensitive to EGCG and LiCl Regulation.** In order to test whether the Wnt signaling in HEK293W cell line was sensitive to be regulated, we treated the cells with (–)-epigallocatechin-3-gallate (EGCG) and lithium chloride (LiCl), a Wnt signaling inhibitor and agonist, respectively. EGCG inhibits the activity of Wnt/β-catenin signaling[14], whereas, LiCl inhibits GSK-3β and subsequently interferes the β-catenin degradation complex, leading to the accumulation of β-catenin in cytoplasm and nucleus. As shown in Figure 2A, EGCG inhibits the activity of Wnt/β-catenin signaling in a dose-dependent manner, and LiCl activated Wnt signaling in a dose- and time-dependent manner in HEK293W cells in Figure 2B. Furthermore, we tested whether tumor chemotherapeutic drugs such as Paclitaxel and Cisplatin (DDP) inhibited Wnt signaling in HEK293W cell line. The Figure 2C showed that neither of them showed obvious effects on the luciferase activity, suggesting the specific activation of Wnt signaling pathway in HEK293W cell line.

To extend our studies to the expression of Wnt target genes which are known tobe regulated by Wnt/β-catenin signaling in mammalian cells, we monitored mRNA expression of Axin2, Cyclin D1 and protein expression of survivin, Cyclin D1[15,16]. The Figures 2D–F showed that EGCG inhibited the expression of these target genes whereas LiCl increased the expression in HEK293W cell line.

**The Nuclear and Cytoplasmic β-Catenin were Increased in HEK293W Cells Compared with HEK293 Cells.** Overall increase of the nuclear and cytoplasmic free β-catenin was further confirmed by immunofluorescence in HEK293W cells, using an anti-β-catenin antibody. The results revealed that the remaining of β-catenin was found to be concentrated in adherence junctions in HEK293 cells (Figure 3, red arrowheads). Compared with HEK293 cells, the active form of β-catenin was increased in the nucleus and free β-catenin was elevated in the cytoplasm in HEK293W cells. Taken together, our study suggests that HEK293W was expressed a robust β-catenin/TCF transcriptional activation which could be monitored easily by the TopFlash reporter.

**Figure 2.** HEK293W cell line was sensitive to EGCG and LiCl regulation of Wnt signaling pathway. (A) EGCG inhibited the activity of Wnt/β-catenin signaling in a dose-dependent manner. (B) LiCl activated Wnt signaling in a dose- and time-dependent manner. Triplicate experiments were done, and the results were expressed as mean ± SD (error bars). Significant inhibitions or activations ($p < 0.05$) were observed in HEK293W cells. TopFlash reporter gene activation is expressed in terms of relative light units (RLU) and normalized with renilla activity. (C) Paclitaxel (taxol) and Cisplatin (DDP) had no effects on luciferase activity in HEK293W cells. (D) Quantitative real-time RT-PCR showed that EGCG inhibited the target genes expression of Wnt signaling and (E) showed LiCl increased the target gene Axin2 expression in HEK293W cells. Triplicate experiments were done, and the results were expressed as mean ± SD (error bars). Statistical significance was determined by paired *t* test. *, $P < 0.05$; **, $P < 0.01$. Significant inhibitions or activations ($p < 0.05$) were observed in HEK293W cells. (F) Western blotting assay showed EGCG inhibited the expression of TCF/β-catenin target genes Cyclin D1 and Survivin in HEK293W cells, with β-actin as loading control.

**Figure 3.** Distribution of β-catenin in HEK293W and HEK293 cells. β-catenin (red arrowheads) was observed present in the nucleus in HEK293W cells whereas β-catenin was not detected in the nucleus and remaining β-catenin was concentrated in adherent junctions (red arrowheads) in HEK293 cells.

**Screening of Inhibitors Targeting the Canonical Wnt Signaling.** To discover small molecular inhibitors targeting the canonical Wnt signaling, we employed reporter assays and screened compounds from a library of natural compounds, which are selected based on the structural diversity, stability, and molecular mass. The initial screening concentration was set from 10 $\mu$M and positive hits were considered when inhibition ratio is higher than 50% (red dotted line in Figure 4). About 2000 compounds were screened in the primary tests, more than 10 compounds exhibited inhibitory effects, and the positive ratio approximately 1%. In the following re-screening tests of the primary hits, the reporter cells were incubated with various concentrations of the compound to estimate the potential inhibitory effects with $IC_{50}$. Among the primary hits, a series of hlsp derivatives exhibited obvious inhibitory activity towards Wnt signaling pathway. In the secondary screening, compounds hlsp-2 and hlsp-19 exhibited significantly inhibitory effect in a dose dependent manner in HEK293W cells, and the $IC_{50}$ value was 0.78 ± 0.18 $\mu$M and 0.33 ± 0.08 $\mu$M, respectively (Figure 5B–C). However, the analogue hlsp-1 showed no effect on Wnt signaling (Figure 5A). These compounds were isolated from the root bark of *Meliaazedarach* and showed obvious cytotoxicity towards tumor cell lines *in vitro*[17]. Furthermore, we analyzed the structure-activity relationship among hlsp-2, hlsp-19 and hlsp-1 (Figures 5D–E). Base on compound hlsp-2 and hlsp-19 exhibited significantly inhibitory effect on Wnt signaling, which only were acetylated individually at C-7 or C-29, whereas hlsp-1 showed markedly decreased inhibitory activity on Wnt signaling, we proposed that existed deacetylation simultaneously at C-7 and C-29 may be responsible for the inhibitory effect on Wnt signaling. We will further explore the activity and the machenisms of these compounds with various Wnt pathway-dependent cancer cell lines.

## Conclusion

Natural products have played a critical role in the field of cancer chemotherapy and numerous marketed drugs are of natural origin, either as derivatives or after modification[18,19]. However, the lack of biological activity and relevant pharmacological research limits their use as drugs for treatment disease. It remains a challenge to demonstrate the mechanism and the target of anti-tumor effect of natural compounds while the cytotoxic profiles can be tested easily.

In the present study we set up a cell-based assay for identifying novel selective inhibitors targeting Wnt signaling pathway by using a Dual-Lucy Assay system[20]. This reporter gene system provides a simple and sensitive assay which can serve as an effective screening model to identify Wnt signaling inhibitors. Combining with the further activity and mechanism study with Wnt-dependant tumor cells, the potential of the inhibitors screened in this study as anti-tumor drug leads targeting the Wnt signaling pathway will be explored.

## Experimental Section

**Materials.** HEK293 cell line was obtained from the American Type Culture Collection (ATCC) and cultured according to supplier's instructions; Lipofectamine 2000 (Invitrogen); Mouse monoclonal anti-β-catenin (BD); Mouse monoclonal anti-Cyclin D1, survivin and β-actin (Santa Cruz Biotechnology); Anti-mouse monoclonal IgG HRP conjugated secondary antibody (Santa Cruz Biotechnology); SuperSignal@ West Pico (Thermo scientific); TopFlash, Renilla reporters and *wnt*3a plasmids with selectable Neo or Hyg were kindly gifted by Dr. Mao (Kunming Institute of Zoology, CAS); Hygromycin B, G418 and EGCG were purchased from Sigma. The library of natural compounds was obtained from the State

**Figure 4.** Primary screening for identifying Wnt antagonists. The concentration of test compounds was set at 10 $\mu$M. The inhibition ratio of relative luciferase activity higher than 50% (red dotted line) relative to the negative control (DMSO) represents positive hits (by red signs).

Key Laboratory of Phytochemistry and Plant Resources in West China.

**Cell Culture and Transfection.** HEK293 cells were propagated in Dulbecco's modified Eagle's medium supplemented with 10% FBS, 100 $\mu$g/mL streptomycin and 100 U/mL penicillin in a humidified atmosphere with 5% $CO_2$. Stable cell line HEK293W was obtained by co-transfection of TopFlash and Renilla and the *wnt*3a into HEK293 cells using Lipofectamine 2000 reagent according to the manufacturer's instructions (Invitrogen). After 24 h of transfection, hygromycin B and G418 were added, and the cells were selected for 14 days. The cell colonies were then detached and reseeded onto 6-well plate. Stable clones were selected for resistance to G418 and hygromycin B. Reporter gene assay was performed to monitor the success of stable transfection.

**TopFlash Reporter Assay.** HEK293W cells were seeded in 96 well plate containing 100 $\mu$L DMEM and 10% FBS. Drugs were added 12 h later and luciferase activities were measured 24 h later, using the Dual-Lucy Assay Kit (Promega) according to the manufacturer's protocol. Luminescence was measured in the Thermo Luminoskan Ascent. Analysis of inhibitors dose-response data and calculation of $IC_{50}$ values were performed using GraphPad Prism (GraphPad Software).

**Real-Time RT-PCR.** Total RNA was extracted from HEK293W cells using Trizol (Invitrogen). First strand cDNA was made using Superscript II reverse transcriptase (Invitrogen) with oligo dT. The Axin2 forward primer was 5'-CTGGAGAGGGAGAAATGCGT-3' and the reverse primer was 5'-ATCTGCTGCTTCTTGATGCCA-3'. The Cyclin D1 forward primer was 5'-AAGTGCGAGGAGGAGGTCTT-3' and the reverse primer was 5'-GGATGGAGTTGTCGGTGTAGA-3'. The β-actin forward primer was 5'-CGCGAGAAGATGACCCAGAT-3' and the reverse primer was 5'-GATAGCACAGCCTGGATAGCAAC-3'. Real-time RT-PCR experiments were performed in triplicate and PCR efficiency with given primers was between 95 and 105%. Melting curves were also performed for identification of primer-specific amplifications. The mRNA expression was normalized with β-actin.

E

| Compounds | $IC_{50}$(μM) |
|---|---|
| hlsp-1 | >10 |
| hlsp-2 | 0.78±0.18 |
| hlsp-19 | 0.33±0.08 |

**Figure 5.** Secondary screening tests of the primary hits. (A) The structures of hlsp-1, hlsp-2, and hlsp-19. (B) The $IC_{50}$ values of hlsp-1, hlsp-2 and hlsp-19 on Wnt signaling. (C–E) hlsp-1, hlsp-2 and hlsp-19 inhibited Wnt signaling in HEK293W cells, and the results were expressed as mean ± SD (error bars).

**Immunocytochemistry.** Cells were fixed with 4% paraformaldehyde and 0.1% Triton X-100 for 20 min at room temperature. The cells were blocked in 3% BSA for 30 min at room temperature and then were incubated with 1:500 dilution of appropriate primary antibody followed by incubation with 1:1000 dilution FITC-conjugated secondary antibody. Cells were analyzed under a fluorescent microscope ECLIPSE Ti-S (Nikon Instech Co. Ltd., Japan).

**Western Blotting.** The cell pellets were re-suspended in lysis buffer (50 mM Tris pH 7.6, 0.2 mM EDTA, 10 mM $MgCl_2$, 0.2% Triton X-100, protease inhibitor cocktail) and homogenized. The homogenate was centrifuged at 12,000 g for 10 min at 4 °C. Protein samples were separated on 10% SDS-PAGE and were transferred to PVDF membranes. The membranes were incubated with mouse monoclonal antibody of Cyclin D1 or surviving at 1:1000 dilution followed by incubation with secondary antibody. Specific proteins were detected using Luminescent Image Analyzer LAS-4000mini system.

**Statistical Analysis.** Statistical analysis was done using independent-sample T-test and the computer SPSS software. In all the assays, the probability value (p) of $< 0.05$ was considered statistically significant.

## Acknowledgments

We thank Dr. B. Mao for TopFlash and renilla plasmids, and Dr. H. Zhou for scientific and technological comments and advice on the manuscript. This project was supported financially by the hundreds top talents program awarded to Yan Li from the Chinese Academy of Sciences, the Major State Basic Research Development Program of China (No. 2009CB522300), the NSFC (No.81173076), and the project of recruited top talent of sciences and technology of Yunnan Province (2009C1120).

**Open Access** This article is distributed under the terms of the Creative Commons Attribution License which permits any use, distribution, and reproduction in any medium, provided the original author(s) and source are credited.

## References

[1] Muller J. M., Chevrier L., Cochaud S., Meunier A. C., Chadeneau C. (2007). Drug Discov. Today Dis. Mech..

[2] Klaus A., Birchmeier W. (2008). Nat. Rev. Cancer.

[3] Clevers H., Nusse R. (2012). Cell.

[4] de Sousa E. M., Vermeulen L., Richel D., Medema J. P. (2011). Clin. Cancer Res..

[5] van Es J. H., Jay P., Gregorieff A., van Gijn M. E., Jonkheer S., Hatzis P., Thiele A., van den Born M., Begthel H., Brabletz T., Taketo M. M., Clevers H. (2005). Nat. Cell Biol..

[6] Brabletz T., Jung A., Dag S., Hlubek F., Kirchner T. (1999). Am. J. Pathol..

[7] Ceballos M. P., Parody J. P., Alvarez Mde L., Ingaramo P. I., Carnovale C. E., Carrillo M. C. (2011). Biochem. Pharmacol..

[8] Wang Y., Krivtsov A. V., Sinha A. U., North T. E., Goessling W., Feng Z., Zon L. I., Armstrong S. A. (2010). Science.

[9] Lu D., Zhao Y., Tawatao R., Cottam H. B., Sen M., Leoni L. M., Kipps T. J., Corr M., Carson D. A. (2004). Proc. Natl. Acad. Sci. U. S. A..

[10] Chien A. J., Moore E. C., Lonsdorf A. S., Kulikauskas R. M., Rothberg B. G., Berger A. J., Major M. B., Hwang S. T., Rimm D. L., Moon R. T. (2009). Proc. Natl. Acad. Sci. U. S. A..

[11] Wang L., Heidt D. G., Lee C. J., Yang H., Logsdon C. D., Zhang L., Fearon E. R., Ljungman M., Simeone D. M. (2009). Cancer Cell.

[12] Vaira S., Friday E., Scott K., Conrad S., Turturro F. (2012). J. Cell Physiol..

[13] Ilyas M., Tomlinson I. P., Rowan A., Pignatelli M., Bodmer W. F. (1997). Proc. Natl. Acad. Sci. U. S. A..

[14] Kim J., Zhang X., Rieger-Christ K. M., Summerhayes I. C., Wazer D. E., Paulson K. E., Yee A. S. (2006). J. Biol. Chem..

[15] Shtutman M., Zhurinsky J., Simcha I., Albanese C., D’Amico M., Pestell R., Ben-Ze’ev A. (1999). Proc. Natl. Acad. Sci. U. S. A..

[16] Tetsu O., McCormick F. (1999). Nature.

[17] Itokawa H., Qiao Z. S., Hirobe C., Takeya K. (1995). Chem. Pharm. Bull..

[18] Cragg G. M., Grothaus P. G., Newman D. J. (2009). Chem. Rev..

[19] Newman D. J., Cragg G. M. (2012). J. Nat. Prod..

[20] Thorne N., Inglese J., Auld D. S. (2010). Chem. Biol..

